# Lifestyle-based oxidative balance score and its association with cardiometabolic health of the community-dwelling elderly: A cross-sectional secondary analysis

**DOI:** 10.3389/fcvm.2022.1000546

**Published:** 2022-09-27

**Authors:** Yang Li, Huixiao Yuan, Qingqing Li, Shasha Geng, Xin Chen, Yingqian Zhu, Hua Jiang

**Affiliations:** ^1^Department of General Practice, Shanghai East Hospital, Tongji University School of Medicine, Shanghai, China; ^2^Department of Geriatrics, Shanghai East Hospital, Tongji University School of Medicine, Shanghai, China

**Keywords:** healthy lifestyle, oxidative stress, cardiometabolic risk factors, community-dwelling elderly, general practice

## Abstract

**Background:**

Cardiometabolic diseases, the main disease burden in older adults, are largely caused by oxidative stress resulting from lifestyle factors. This study investigated the relationship between lifestyle-based oxidative balance scores and cardiometabolic health among the community-dwelling elderly.

**Methods:**

This work conducted a secondary analysis of previous cross-sectional research data and constructed a lifestyle-based oxidative balance score (LOBS) including 4 components (higher scores were considered more antioxidant). Linear regression models and logistic regression models were used to evaluate the associations with cardiometabolic biomarkers and the number of cardiometabolic risk factors. Besides, we investigated whether these associations differed by covariates.

**Results:**

A total of 710 individuals (60.99% female, median age 70.0 years) were recruited. The inverse associations of LOBS with SBP and TG and the positive association with HDLC were statistically significant in both linear and logistic regression models. In contrast, an inverse association of LOBS with DBP was significant only in the linear regression model (all *P* < 0.05). The associations of LOBS with TG and HDLC were not affected by age, gender, or socioeconomic level. A significant inverse association was observed between LOBS and the number of cardiometabolic risk factors. Compared with the lowest LOBS, the ORs for more cardiometabolic risk factors in the second and third intervals were 0.577 (0.422, 0.788) and 0.460 (0.301, 0.703) (both *P* < 0.001).

**Conclusion:**

In summary, this study shows that antioxidant-predominant lifestyle exposure yields a better cardiometabolic health status. We recommend that general practitioners should offer comprehensive healthy lifestyle management to community-dwelling elderly.

## Introduction

China has the largest elderly population in the world. According to the seventh national census in 2020, 264 million people are aged 60 or above, accounting for about 18.7% of the total population (1). As a result, aging-related diseases present a significant challenge to the healthcare system in China.

The major causes of disease burden among the Chinese elderly are cardiometabolic diseases (CMD) including hypertension, diabetes, dyslipidemia, ischemic heart disease, stroke, and chronic kidney disease (2). The development of CMDs is characterized by a long prodromal period, in which clinical symptoms become apparent only after considerable impairment. While abnormalities in cardiometabolic biomarkers (e.g., high total cholesterol), as downstream secondary lesions, might inaccurately reflect the early stages of chronic subclinical processes (3). Evidence suggests that oxidative stress-related biochemical changes contribute to the onset and progression of CMD, including diabetes (4), hypertension (5), and atherosclerosis (6). As such, incorporating oxidative stress measurements into risk assessment tools can improve the predictive and prognostic value of conventional risk factors.

The main causes of CMD include unhealthy lifestyle factors with high pro-oxidant and low antioxidant capacity (7). Continued exposure to unhealthy lifestyles significantly increases oxidative stress and the risk of chronic cellular damage. Moreover, the accumulation of cellular damage may induce pathological phenotypes (7). Therefore, it is important to investigate how lifestyle behaviors affect oxidative balance to reveal strategies for predicting future trends in health or disease.

There is a possibility of complex interactions among lifestyle factors [e.g., smoking (8), alcohol consumption (9), overweight/obesity (10), diet (11), and physical activity (12)]. The oxidative balance scores (OBS) (13, 14) incorporating multiple dietary and lifestyle exposures have been developed to show the collective oxidative effects and examine the relationship between oxidative status and the risk of chronic diseases (15). To date, few studies have investigated the correlation between oxidative stress and cardiometabolic health among the community-dwelling elderly.

Therefore, we constructed a lifestyle-based OBS tool that can evaluate the oxidative balance status of an individual. Subsequently, we investigated the associations between lifestyle-based OBS and the number of cardiometabolic risk factors (CMRF) (16) as well as various cardiometabolic biomarkers, including systolic blood pressure (SBP), diastolic blood pressure (DBP), fasting plasma glucose (FPG), triglycerides (TG), total cholesterol (TC), high-density lipoprotein cholesterol (HDL-C) and low-density lipoprotein cholesterol (LDL-C). Additionally, we examined whether these associations differed by covariates.

## Methods

### Study design and participants

Based on a completed cross-sectional study, this secondary analysis assessed the prevalence of frailty among community-dwelling elderly in Pudong New Area, Shanghai. Participants were recruited through two-stage sampling between May and August 2019. First, Pudong New Area was divided into low-level and high-level regions as per the level of social-economic status. Thereafter, based on the sample size of the elderly population and the approval of the community health centers (CHCs), four communities were selected from each region, totaling eight communities. Community residents aged 60 and above who were willing to provide informed consent were invited to their preferred CHCs for data collection and frailty phenotype assessment. Exclusion criteria were factors that could directly affect the frailty assessment including (1) prior hospitalization within the last 6 months, (2) severe organ failure, (3) malignant tumor, (4) severe physical disabilities, (4) severe mental illness, (5) severe cognitive impairment or dementia.

**Figure 1:**
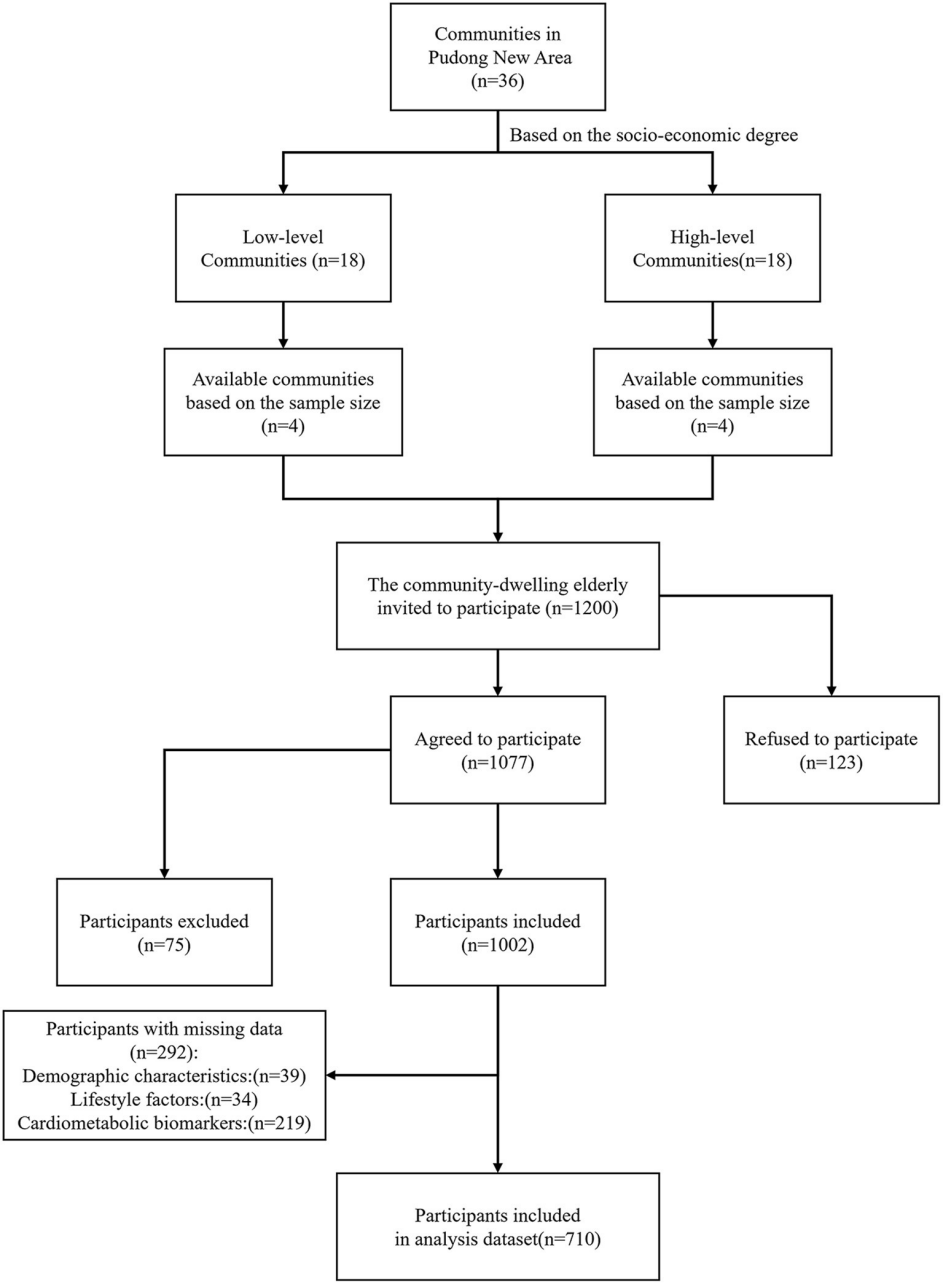
Flowchart of participants included in the secondary analysis.

Among all the 1002 individuals who were recruited consecutively in this cross-sectional study, 39 participants without demographic characteristics, 34 participants without data on the lifestyle-based OBS components, and 219 participants who had no or missing data on 7 cardiometabolic biomarkers were excluded. Therefore, 710 individuals with complete data were included for analysis (Figure 1).

### Lifestyle-based OBS (LOBS) components and assessments

Information on lifestyle-based oxidative balance score (LOBS) components was collected using a lifestyle questionnaire. The questionnaire included physical activity with indirect antioxidant capacity, as well as smoking, alcohol consumption, and overweight/obesity with pro-oxidant capacity. All variables were grouped into three levels and assigned 0, 1, or 2 values, respectively.

Physical activity: The physical activity level of participants was evaluated based on the self-reported frequency of moderate and vigorous activity per week and then divided into 3 ranks: low (moderate or vigorous activity less than once per week), moderate (vigorous activity once per week plus moderate activity once per week, or moderate activity 2–4 times per week), and high (vigorous activity twice per week or moderate activity more than 4 times per week). Each moderate or vigorous activity should be no <40 min and the shortfall should be calculated proportionally.

Smoking: According to their self-report, participants were classified as never-smokers, former smokers, and current smokers.

Alcohol consumption: Based on self-reported and gender differences, participants were categorized as non-drinkers, moderate drinkers (1–7 drinks per week for women and 1–14 drinks per week for men), and heavy drinkers (more than 7 drinks per week for women and 14 drinks per week for men).

Overweight/obesity: Body mass index (BMI) was calculated as weight divided by height squared (kg/m^2^). According to the recommended standards of China Health Management Society (17), all participants were categorized as underweight/normal (BMI <24.0 kg/m^2^), overweight (24.0 ≤ BMI <28.0kg/m^2^), and obesity (BMI≥28.0 kg/m^2^).

In previous studies (13, 14), the multicomponent OBSs were created using different questionnaires and successfully validated through their relationship with circulating F2-isoprostanes concentrations. Consequently, the associations between OBS and health outcomes were comparable regardless of the weighting methods used to create OBS (equal-weighted, literature review-derived, study data-based, and Bayesian methods) (13, 14). In this study, the initial value of each component was multiplied by +1 or –1 for antioxidant or pro-oxidant, respectively, as the component weights of LOBS (Table 1). LOBS was calculated by combining the scores of each component. High LOBS indicated a possible beneficial balance between pro-oxidants and antioxidants. The correlation of LOBS with WBC and NEUT has been verified. Detailed results are available in the Supplementary Table 1.

**Table 1:** Components and their weights of lifestyle-based oxidative balance scores (LOBS).

**Component**	**Level**	**Definition**	**Weight score**
**Lifestyle antioxidants**			
Physical activity	High	Vigorous activity twice per week or moderate activity more than 4 times per week	+ 2
	Moderate	Vigorous activity once per week plus moderate activity once per week, or moderate activity 2-4 times per week	+ 1
	Low	Moderate or vigorous activity less than once per week	
**Lifestyle prooxidants**			
Smoking	Current	Currently smokes tobacco	– 2
	Former	Used to smoke	– 1
	Never	Never smoked	
Alcohol consumption	Heavy	Woman:> 7 drinks per week	– 2
		Man:> 14 drinks per week	
	Moderate	Woman:1–7 drinks per week	– 1
		Man:1–14 drinks per week	
	Never	No alcohol consumption	
Overweight/Obesity	Obesity	BMI ≥ 28.0 kg/m^2^	– 2
	Overweight	24.0 ≤ BMI <28.0kg/m^2^	– 1
	Underweight/normal	BMI <24.00 kg/m^2^	

### Definition of cardiometabolic risk factors

Three types of CMRFs were considered in this study, including hypertension, diabetes and dyslipidemia. A lower number of CMRFs implied better cardiometabolic health.

Three blood pressure measurements on participants were performed using a standardized electronic sphygmomanometer, recording systolic blood pressure (SBP) and diastolic blood pressure (DBP). Hypertension was defined as SBP ≥140 mmHg or DBP≥90 mmHg or the use of any antihypertensive medication (18).

After fasting for more than 10 h, blood samples were collected from all participants. Cobas8000^®^ modular analyzer series (Basel, Switzerland) was used to measure the fasting blood glucose (FPG), triglyceride (TG), total cholesterol (TC), high-density lipoprotein cholesterol (HDLC), and low-density lipoprotein cholesterol (LDLC) by standard methods. Type 2 diabetes mellitus (T2DM) was defined by the American Diabetes Association guidelines (19) as FPG ≥ 7.0 mmol/L or the use of any glucose-lowering medication. Dyslipidemia was defined as TG ≥ 2.3 mmol/L, TC ≥ 6.2 mmol/L, LDL-C ≥ 4.1 mmol /L, HDL-C < 1.0 mmol/L, or the use of any hypolipidemic drugs based on Chinese guidelines for the management of dyslipidemia in adults dyslipidemia (20).

### Covariates

Based on previously published literature and biological justification, covariates for this study included self-reported age, gender, educational degree (primary school or less, middle school, high school, college or higher), solitary status (yes or no), and socioeconomic level of communities (previously encoded low-level and high-level).

### Statistical analysis

#### Primary analysis

In the descriptive analysis, characteristics of the study population were reported overall and across LOBS intervals. For continuous variables, the Shapiro-Wilk test was used to test for normality distribution. Analysis of variance (ANOVA) was used for continuous or ordinal categorical variables with a normal distribution; the Kruskal-Wallis test was performed for those with a skewed distribution. The Chi-square test was used to analyze dichotomous variables and to examine differences in characteristics across LOBS intervals.

LOBS was the independent variable of interest considered a continuous or categorical variable. LOBS was grouped by tertile intervals, with the first LOBS interval representing the preponderance of pro-oxidants as a reference. The dependent variables of interest were the number of CMRFs and cardiometabolic biomarkers which included systolic blood pressure (SBP), diastolic blood pressure (DBP), fasting plasma glucose (FPG), triglycerides (TG), total cholesterol (TC), high-density lipoprotein cholesterol (HDL-C), and low-density lipoprotein cholesterol (LDL-C). Multivariate linear regression models were constructed to assess the relationships of LOBS with cardiometabolic biomarkers and CMRF numbers.

Associations of LOBS with abnormal biomarkers and CMRF numbers were calculated using multivariate logistic regression models. Results were expressed as adjusted odds ratios (ORs) and their corresponding 95% confidence intervals (CIs), adjusted for age, gender, educational degree, solitary situation, and socio-economic level of the community. The covariance among the independent variables in all models was also tested.

#### Stratified analysis

Statistical differences between models with or without interaction terms (LOBS^*^covariates) were evaluated using likelihood ratio tests to establish the effect of covariates on these associations. Stratified analyses were performed by age ( ≤ /> median age of 70 years), gender (male/female), and socioeconomic status of communities (low-level and high-level). The solitary status was excluded since the conditions for stratified analysis were lacking.

#### Sensitivity analysis

Sensitivity analysis was performed to investigate the effects of individual components by eliminating each component from the LOBS and controlling it as a covariate. We also assessed the impact of different LOBS classification methods (grouping by quartiles) on the outcomes.

All statistical analyses were performed using the STATA software version 16.0 for Windows (Stata Corporation, College Station, TX). All *P*-values were two-sided. *P*-values < 0.05 or 95% CI, excluding 1.0 were considered statistically significant.

## Results

### Participant characteristics

A total of 710 participants were recruited for this study. The mean age (interquartile range) of the participants was 70.0 (67.0–75.0) years, and 60.99% were female (Table 2). Among the participants, 58.31% had hypertension, 13.94% had diabetes mellitus, and 38.73% had dyslipidemia. Only 24.37% of the participants did not have any of these three diseases, whereas 30.99% concurrently had more than two CMRFs.

**Table 2:** The characteristics of selected participants according to lifestyle-based oxidative balance scores quartiles.

	**All (*n* = 710)**	**LOBS**^**a**^ **intervals**			
		**T1 (*n* = 290)**	**T2 (*n* = 312)**	**T3 (*n* = 108)**	***P*-value**
**Sociodemographic features**					
Age (years), Median (IQR)	70.0 (67.0–75.0)	70.0 (66.0,75.0)	70.0 (67.0–75.0)	70.5 (67.0–75.5)	0.634
Gender, *n* (%)					<0.001
Male	277 (39.01)	163 (56.21)	87 (27.88)	27 (25.00)	
Female	433 (60.99)	127 (43.79)	225 (72.12)	81 (75.00)	
Educational degree, *n* (%)					0.357
Primary school or less	196 (27.61)	71 (24.48)	96 (30.77)	29 (26.85)	
Middle school	277 (39.01)	113 (38.97)	120 (38.46)	44 (40.74)	
High school	139 (19.58)	65 (22.41)	58 (18.59)	16 (14.81)	
College or higher	98 (13.80)	41 (14.14)	38 (12.18)	19 (17.59)	
Solitary status, *n* (%)					0.840
Yes	82 (11.45)	34 (11.72)	34 (10.90)	14 (12.96)	
No	628 (88.55)	256 (88.28)	278 (89.10)	94 (87.04)	
Socio-economic degree, *n* (%)					0.076
Low-level	268 (37.75)	97 (33.45)	132 (42.31)	39 (36.11)	
High-level	442 (62.25)	193 (66.55)	180 (57.69)	69 (63.89)	
**Cardiometabolic Biomarkers**					
SBP (mmHg), Mean (SD)	142.8 (18.9)	144.4 (17.9)	142.1 (20.0)	140.3 (17.8)	0.109
DBP (mmHg), Median (IQR)	79.0 (72.0–86.0)	80.0 (73.0–87.0)	78.0 (70.5–85.0)	74.5 (68.0–84.0)	0.002
FPG (mmol/L), Median (IQR)	5.30 (4.90–6.00)	5.36 (4.94–6.10)	5.22 (4.89–5.89)	5.20 (4.99–5.70)	0.132
TG (mmol/L), Median (IQR)	1.50 (1.09–2.00)	1.62 (1.16–2.12)	1.49 (1.07–1.96)	1.26 (0.91–1.73)	<0.001
TC (mmol/L), Median (IQR)	5.00 (4.39–5.62)	4.90 (4.35–5.58)	5.00 (4.38–5.68)	5.11 (4.43–5.78)	0.410
HDLC (mmol/L), Median (IQR)	1.27 (1.06–1.54)	1.19 (1.02–1.44)	1.31 (1.12–1.56)	1.44 (1.20–1.70)	<0.001
LDLC (mmol/L), Median (IQR)	2.89 (2.31–3.43)	2.88 (2.30–3.42)	2.91 (2.33–3.43)	2.84 (2.34-3.43)	0.890
Hypertension, *n* (%)	414 (58.31)	186 (64.14)	168 (53.85)	60 (55.56)	0.031
Diabetes, *n* (%)	99 (13.94)	44 (15.17)	44 (14.10)	11 (10.19)	0.440
Dyslipidemia, *n* (%)	275 (38.73)	130 (44.83)	116 (37.18)	29 (26.85)	0.004
**Number of CMRFs**					0.004
0	173 (24.37)	50 (17.24)	89 (28.53)	34 (31.48)	
1	317 (44.65)	136 (46.90)	133 (42.63)	48 (44.44)	
2	189 (26.62)	88 (30.34)	75 (24.04)	26 (24.07)	
3	31 (4.37)	16 (5.52)	15 (4.81)	/	
**LOBS components**					/
Physical activity, *n* (%)					
High	274 (38.59)	22 (7.59)	144 (46.15)	108 (100.00)	
Moderate	86 (12.11)	30 (10.34)	56 (17.95)	/	
Smoking, *n* (%)					
Current	67 (9.44)	63 (21.72)	4 (1.28)	/	
Former	53 (7.46)	47 (16.21)	6 (1.92)	/	
Alcohol consumption, *n* (%)					
Heavy	20 (2.82)	20 (6.90)	/	/	
Moderate	49 (6.90)	37 (12.76)	12 (3.85)	/	
BMI Level, *n* (%)					
Obesity	109 (15.35)	81 (27.93)	28 (8.97)	/	
Overweight	304 (42.82)	171 (58.97)	133 (42.63)	/	

SBP, systolic blood pressure; DBP, diastolic blood pressure; FPG, fasting plasma glucose; TG, triglycerides; TC, total cholesterol; HDLC, high-density lipoprotein cholesterol; LDLC, low-density lipoprotein cholesterol; CMRF, cardiometabolic risk factors.

^a^Grouped by tertile intervals, the first LOBS interval representing the preponderance of pro-oxidants was used as a reference. Tertile 1, LOBS−6~-1; Tertile 2, LOBS 0~1; Tertile 3, LOBS 2.

The LOBS ranged between –6 and 2 with a mean of –0.23 (standard deviation: 1.55). Unlike the lowest LOBS interval (range –6 to –1), a greater proportion of participants at the highest LOBS interval (range 2) were female (Table 2). Notably, HDLC levels increased whereas TG levels and the proportion of participants with dyslipidemia decreased with the increase of LOBS.

### Primary analysis

Table 3-1 shows the associations of LOBS (continuous variable) with each cardiovascular metabolic biomarker. The adjusted multivariate linear regression model results revealed that the associations of LOBS with SBP, DBP, TG, and HDLC were statistically significant. Analysis of regression coefficients revealed that SBP decreased by 0.999 mmHg (95% CI: –1.917, –0.080; *P* = 0.033), TG decreased by 0.100 mmol/L (95% CI: –0.154, –0.046; *P* < 0.001), whereas HDLC increased by 0.034 mmol/L (95% CI: 0.015, 0.053; *P* < 0.001) for each unit increase in LOBS. In comparison with the first interval of LOBS, SBP of participants decreased by 3.509 mmHg (95% CI: –6.518, –0.500; *P* < 0.05) and 5.107 mmHg (95% CI: –9.210, – 1.004; *P* < 0.05), DBP decreased by 2.108 mmHg (95% CI: –3.907, –0.309; *P* < 0.05) and 2.590 mmHg (95% CI: –5.043, –0.138; *P* < 0.05), TG decreased by 0.214 mmol/L (95% CI: –0.391, –0.037; *P* < 0.05) and 0.357 mmol/L (95% CI: –0.598, –0.115; *P* < 0.01), and HDLC increased by 0.093 mmol/L (95% CI: 0.030, 0.156; *P* < 0.01) and 0.163 mmol/L (95% CI: 0.077, 0.248; *P* < 0.001), respectively in the 2nd and 3rd intervals. Overall, there was a significant dose-response relationship between LOBS and SBP (*P* trend = 0.006), DBP (*P* trend = 0.015), TG (*P* trend = 0.002), and HDLC (*P* trend<0.001).

**Table 3-1:** Associations of the lifestyle-based oxidative balance score (tertile intervals) with the number of CMRFs and cardiometabolic biomarkers using linear regression.

	**SBP**	**DBP**	**FPG**	**TG**	**TC**	**HDLC**	**LDLC**	**Number of CMRFs**
LOBS ^a^	– 0.999	– 0.242	– 0.052	– 0.100	– 0.030	0.034	– 0.019	– 0.089
95% CI	– 1.917, – 0.080	– 0.792, 0.309	– 0.141, 0.037	– 0.154, – 0.046	– 0.080, 0.020	0.015, 0.053	– 0.063, 0.024	– 0.131, – 0.048
*P*-value	0.033	0.389	0.249	<0.001	0.237	<0.001	0.386	<0.001
Tertile 1^b^	/	/	/	/	/	/	/	/
Tertile 2	– 3.509*	– 2.108*	– 0.098	– 0.214*	– 0.027	0.093**	– 0.005	– 0.230***
95% CI	– 6.518, – 0.500	– 3.907, – 0.309	– 0.389, 0.194	– 0.391, – 0.037	– 0.191, 0.137	0.030, 0.156	– 0.148, 0.139	– 0.365, – 0.094
Tertile 3	– 5.107*	– 2.590*	– 0.110	– 0.357**	– 0.026	0.163***	– 0.052	– 0.347***
95% CI	– 9.210, – 1.004	– 5.043, – 0.138	– 0.508, 0.287	– 0.598, – 0.115	– 0.249, 0.198	0.077, 0.248	– 0.248, 0.144	– 0.531, – 0.162
P _trend_	0.006	0.015	0.512	0.002	0.774	<0.001	0.650	<0.001

SBP, systolic blood pressure; DBP, diastolic blood pressure; FPG, fasting plasma glucose; TG, triglycerides; TC, total cholesterol; HDLC, high-density lipoprotein cholesterol; LDLC, low-density lipoprotein cholesterol; CMRF, cardiometabolic risk factors; CI, Confidence interval.

^a^Regression coefficients and 95% CIs from multivariate linear regression models. Adjusted for age, gender, educational degree, solitary status, and socio-economic degree of communities to which selected participants belonged.

^b^Grouped by tertile intervals, the first LOBS interval representing the preponderance of pro-oxidants was used as a reference. Tertile 1, LOBS−6~-1; Tertile 2, LOBS 0~1; Tertile 3, LOBS 2. *, **, and *** respectively indicate *P* values < 0.05, < 0.01, and < 0.001.

Table 3-2 presents the associations of LOBS with abnormal levels of cardiometabolic biomarkers. Multivariate logistic regression models revealed that LOBS statistically and significantly correlated with abnormal SBP, TG, and HDLC (all *P* < 0.05). Participants in other LOBS intervals had significantly lower odds of abnormal SBP and HDLC (both *P* trend < 0.05) unlike that in the lowest LOBS, with the odds of abnormal SBP and abnormal HDLC reduced by ~44 and 39%, respectively, in the second interval participants.

**Table 3-2:** Associations of the lifestyle-based oxidative balance score (tertile intervals) with the number of CMRFs and cardiometabolic biomarkers using logistic regression.

	**SBP^c^**	**DBP^c^**	**FPG^d^**	**TG^e^**	**TC^e^**	**HDLC^e^**	**LDLC^e^**	**Number of CMRFs**
LOBS^a^	0.889	1.062	0.908	0.840	0.863	0.861	0.880	0.811
95% CI	0.798, 0.990	0.922, 1.222	0.784, 1.051	0.731, 0.966	0.728, 1.022	0.756, 0.981	0.724, 1.070	0.737, 0.893
*P*-value	0.032	0.403	0.196	0.015	0.088	0.024	0.201	<0.001
Tertile 1^b^	/	/	/	/	/	/	/	/
Tertile 2	0.558**	0.696	0.935	0.738	0.857	0.614*	0.806	0.577***
95% CI	0.391, 0.797	0.436, 1.111	0.582, 1.504	0.474, 1.148	0.515, 1.428	0.395, 0.956	0.447, 1.453	0.422, 0.788
Tertile 3	0.624	0.919	0.674	0.549	0.570	0.553	0.554	0.460***
95% CI	0.386, 1.009	0.491, 1.717	0.327, 1.386	0.285, 1.057	0.270, 1.206	0.286, 1.070	0.227, 1.353	0.301, 0.703
P _trend_	0.010	0.466	0.331	0.052	0.156	0.023	0.188	<0.001

SBP, systolic blood pressure; DBP, diastolic blood pressure; FPG, fasting plasma glucose; TG, triglycerides; TC, total cholesterol; HDLC, high-density lipoprotein cholesterol; LDLC, low-density lipoprotein cholesterol; CMRF, cardiometabolic risk factors; CI, Confidence interval.

^a^Odds ratios and 95% CIs from multivariate logistic regression models. Adjusted for age, gender, educational degree, solitary status, and socio-economic degree of communities to which selected participants belonged.

^b^Grouped by tertile intervals, the first LOBS interval representing the preponderance of pro-oxidants was used as a reference. Tertile 1, LOBS−6~-1; Tertile 2, LOBS 0~1; Tertile 3, LOBS 2.

^c^Blood pressure cutoffs: normal SBP, <140 mmHg, abnormal SBP, ≥140 mmHg; normal DBP, <90 mmHg, abnormal DBP, ≥90 mmHg.

^d^Fasting plasma glucose cutoffs: normal FPG, <7.0 mmol/L, abnormal FPG, ≥7.0 mmol/L.

^e^Lipids/lipoproteins cutoffs: normal TG, <2.3 mmol/L, abnormal TG, ≥2.3 mmol/L; normal TC, <6.2 mmol/L, abnormal TC, ≥6.2 mmol/L; normal HDLC, >1.02 mmol/L, abnormal HDLC, ≤ 1.0 mmol/L; normal LDLC, <4.1 mmol/L, abnormal LDLC, ≥4.1 mmol/L. *, **, and *** respectively indicate *P* values <0.05, <0.01, and <0.001.

As shown in Tables 3-1,3-2, both linear and logistic regression models showed a significant negative relationship between LOBS and the number of CMRFs (both *P* < 0.001). In contrast with the first LOBS, the odds of more CMRFs in the second and third intervals decreased by an estimated 42 and 54% (both *P* < 0.001), with a statistically significant inverse dose-response relationship (*P* trend < 0.001).

### Stratified analysis

Stratified analyses were performed based on selected participant characteristics i.e., age ( ≤ /> median age 70 years), gender (male/female), and socioeconomic levels (low-level/high-level).

The relationship between the continuous variable LOBS and HDLC (*P* = 0.026) was observed only in the lower age groups (Table 4-1). The inverse associations of LOBS with DBP (*P* trend = 0.018) and TG (*P* trend = 0.036) and the positive association with HDLC (*P* trend < 0.001) were observed in the cross-interval comparisons. Besides, LOBS demonstrated a statistically significant negative dose-response relationship with HDLC (both *P* < 0.05). In contrast, significant associations of LOBS with TG and HDLC were observed in the upper age groups, both as continuous variables and intervals, but not between LOBS and DBP. In the adjusted logistic regression model (Table 4-2), significant associations between LOBS and decreased odds of abnormal SBP, FPG, TG, and HDLC were observed only in the upper age group (all *P* < 0.05). However, further comparison across intervals showed a decreasing trend in the odds of abnormal SBP, TG, and HDLC among participants with increased LOBS intervals (all *P* trend < 0.05), including a statistically significant OR for abnormal SBP in the second interval compared to the first interval. The cross-interval analyses in the lower age group revealed an inverse association between LOBS and the number of CMRFs (both *P* trend < 0.05). The OR of the third interval compared to the first interval was statistically significant (*P* < 0.05). However, the negative relationship between LOBS and the number of CMRFs was statistically significant in both linear and logistic regression models in the upper age group (both *P* < 0.001). Compared with the first LOBS, both the second and third intervals had more than 55% lower odds of more CMRFs (both *P* < 0.01), hence a significant inverse dose-response effect (*P* trend < 0.001). Additionally, the interaction between age and LOBS was only more significant in the logistic regression model for abnormal SBP (*P* interaction = 0.041).

**Table 4-1:** Associations of the lifestyle-based oxidative balance score with the number of cardiometabolic risk factors and cardiometabolic biomarkers by age using linear regression.

	**SBP**	**DBP**	**FPG**	**TG**	**TC**	**HDLC**	**LDLC**	**Number of CMRFs**
**Age ≤70 years(*****n*** **= 372)**								
LOBS^a^	– 0.559	– 0.304	0.032	– 0.050	– 0.046	0.032	– 0.050	– 0.049
95% CI	– 1.876, 0.758	– 1.096, 0.488	– 0.076, 0.141	– 0.110, 0.010	– 0.120, 0.028	0.004, 0.061	– 0.112, 0.012	– 0.107, 0.009
*P*-value	0.404	0.451	0.561	0.100	0.221	0.026	0.116	0.097
Tertile 1^b^	/	/	/	/	/	/	/	/
Tertile 2	– 2.397	– 2.107	0.108	– 0.173	0.014	0.104*	0.015	– 0.102
95% CI	– 6.752, 1.959	– 4.712, 0.499	– 0.251, 0.468	– 0.372, 0.025	– 0.231, 0.260	0.010, 0.197	– 0.190, 0.221	– 0.292, 0.089
Tertile 3	– 4.172	– 4.097*	0.022	– 0.262	– 0.144	0.216***	– 0.254	– 0.272*
95% CI	– 10.114, 1.770	−0.7.652, – 0.542	– 0.469, 0.512	– 0.533, 0.008	– 0.479, 0.191	0.088, 0.343	– 0.534, 0.026	– 0.532, – 0.011
P _trend_	0.140	0.018	0.800	0.036	0.504	<0.001	0.152	0.043
**Age > 70 years (*****n*** **= 338)**								
LOBS ^a^	– 1.243	– 0.180	– 0.136	– 0.161	– 0.015	0.036	0.013	– 0.131
95% CI	– 2.554, 0.068	– 0.946, 0.587	– 0.279, 0.07	– 0.252, – 0.069	– 0.081, 0.051	0.011, 0.062	– 0.048, 0.074	– 0.190, – 0.072
*P* value	0.063	0.645	0.063	<0.001	0.653	0.006	0.670	<0.001
Tertile 1^b^	/	/	/	/	/	/	/	/
Tertile 2	– 3.784	– 2.099	– 0.269	– 0.278	– 0.050	0.091*	– 0.008	– 0.345***
95% CI	– 8.067, 0.499	– 4.597, 0399	– 0.738, 0.200	– 0.580, 0.024	– 0.265, 0.166	0.006, 0.176	– 0.207, 0.191	– 0.539, – 0.150
Tertile 3	– 4.957	– 0.953	– 0.256	– 0.483*	0.045	0.101	0.123	– 0.418**
95% CI	– 10.782, 0.868	– 4.351, 2.444	– 0.894, 0.382	– 0.894, – 0.073	– 0.248, 0.338	– 0.014, 0.216	– 0.148, 0.394	– 0.682, – 0.153
P _trend_	0.053	0.329	0.312	0.013	0.914	0.037	0.475	<0.001
P _interaction_	0.150	0.835	0.790	0.701	0.315	0.812	0.283	0.224

SBP, systolic blood pressure; DBP, diastolic blood pressure; FPG, fasting plasma glucose; TG, triglycerides; TC, total cholesterol; HDLC, high-density lipoprotein cholesterol; LDLC, low-density lipoprotein cholesterol; CMRF, cardiometabolic risk factors; CI, Confidence interval.

^a^Regression coefficients and 95% CIs from multivariate linear regression models. Adjusted for gender, educational degree, solitary status, and socio-economic degree of communities to which selected participants belonged.

^b^Grouped by tertile intervals, the first LOBS interval representing the preponderance of pro-oxidants was used as a reference. Tertile 1, LOBS−6~-1; Tertile 2, LOBS 0~1; Tertile 3, LOBS 2.

^c^P _interaction_ from stratified risk factor^*^LOBS interaction term in multivariate linear regression models. *, **, and *** respectively indicate *P* values < 0.05, < 0.01, and < 0.001.

**Table 4-2:** Associations of the lifestyle-based oxidative balance score with the number of cardiometabolic risk factors and cardiometabolic biomarkers by age using logistic regression.

	**SBP^c^**	**DBP^c^**	**FPG^d^**	**TG^e^**	**TC^e^**	**HDLC^e^**	**LDLC^e^**	**Number of CMRFs**
**Age ≤70 years (*****n*** **= 372)**
LOBS ^a^	0.946	1.109	1.108	0.958	0.823	0.939	0.848	0.884
95% CI	0.816, 1.097	0.910, 1.351	0.885, 1.386	0.785, 1.170	0.668, 1.014	0.780, 1.131	0.660, 1.088	0.772, 1.012
*P*-value	0.461	0.304	0.372	0.676	0.068	0.510	0.195	0.074
Tertile 1^b^	/	/	/	/	/	/	/	/
Tertile 2	0.744	0.661	1.490	0.897	0.927	0.610	0.794	0.758
95% CI	0.456, 1.216	0.339, 1.288	0.703, 3.158	0.478, 1.686	0.491, 1.752	0.319, 1.167	0.375, 1.681	0.488, 1.176
Tertile 3	0.774	0.816	1.068	0.681	0.361	0.791	0.321	0.514*
95% CI	0.396, 1.512	0.326, 2.039	0.355, 3.213	0.275, 1.685	0.125, 1.043	0.324, 1.932	0.086, 1.195	0.279, 0.946
P _trend_	0.326	0.437	0.333	0.425	0.097	0.335	0.101	0.031*
**Age > 70 years (*****n*** **= 338)**
LOBS^a^	0.834	1.029	0.776	0.715	0.910	0.794	0.898	0.744
95% CI	0.711, 0.978	0.837, 1.266	0.632, 0.952	0.584, 0.875	0.674, 1.229	0.657, 0.958	0.653, 1.233	0.649, 0.853
*P* value	0.026	0.783	0.015	0.001	0.539	0.016	0.506	<0.001
Tertile 1^b^	/	/	/	/	/	/	/	/
Tertile 2	0.410***	0.771	0.728	0.565	0.725	0.613	0.797	0.442***
95% CI	0.241, 0.696	0.394, 1.507	0.383, 1.384	0.301, 1.060	0.291, 1.805	0.330, 1.136	0.296, 2.142	0.282, 0.693
Tertile 3	0.519	1.053	0.477	0.404	0.826	0.397	0.854	0.401**
95% CI	0.256, 1.051	0.438, 2.534	0.179, 1.270	0.154, 1.060	0.266, 2.571	0.142, 1.109	0.235, 3.098	0.221, 0.727
P _trend_	0.013	0.881	0.650	0.029	0.664	0.038	0.742	<0.001
P ${}_{\text{interaction}}^{\text{f}}$	0.041	0.772	0.380	0.439	0.095	0.648	0.571	0.368

SBP, systolic blood pressure; DBP, diastolic blood pressure; FPG, fasting plasma glucose; TG, triglycerides;TC, total cholesterol; HDLC, high-density lipoprotein cholesterol; LDLC, low-density lipoprotein cholesterol; CMRF, cardiometabolic risk factors; CI, Confidence interval.

^a^Odds ratios and 95% CIs from multivariate logistic regression models. Adjusted for gender, educational degree, solitary status, and socio-economic degree of communities to which selected participants belonged.

^b^Grouped by tertile intervals, the first LOBS interval representing the preponderance of pro-oxidants was used as a reference. Tertile 1, LOBS−6~-1; Tertile 2, LOBS 0~1; Tertile 3, LOBS 2.

^c^Blood pressure cutoffs: normal SBP, <140 mmHg, abnormal SBP, ≥140 mmHg; normal DBP, <90 mmHg, abnormal DBP, ≥90 mmHg.

^d^Fasting plasma glucose cutoffs: normal FPG, <7.0 mmol/L, abnormal FPG, ≥7.0 mmol/L.

^e^Lipids/lipoproteins cutoffs: normal TG, <2.3 mmol/L, abnormal TG, ≥2.3 mmol/L; normal TC, <6.2 mmol/L, abnormal TC, ≥6.2 mmol/L; normal HDLC, >1.02 mmol/L, abnormal HDLC, ≤ 1.0 mmol/L; normal LDLC, <4.1 mmol/L, abnormal LDLC, ≥4.1 mmol/L.

^f^P _interaction_ from stratified risk factor^*^LOBS interaction term in multivariate logistic regression models.*, **, and *** respectively indicate *P* values < 0.05, < 0.01, and < 0.001.

In the linear regression model (Table 5-1), the associations of LOBS with TG, HDLC, and the number of CMRFs were statistically significant (all *P* < 0.01), both as continuous variables and intervals, and showed no difference by gender. A negative association between LOBS and SBP was only detected in women, with a decrease in SBP of 1.578 mmHg (95% CI: –3.030, –0.126; *P* = 0.033) per unit increase in LOBS. In logistic regression models (Table 5-2), the inverse associations of LOBS with abnormal TG (*P* = 0.024) and HDLC (*P* = 0.030) were only found in men; besides, the inverse association of LOBS with abnormal SBP was only observed in women (*P* = 0.029). None of the comparisons across intervals revealed a significant dose-response relationship. The OR of abnormal SBP was statistically significant (*P* < 0.01) only for the second LOBS interval compared to the first. Interestingly, the significant inverse dose-response relationship between LOBS and the odds of more CMRFs was noted in both men and women (both *P* trend < 0.01).

**Table 5-1:** Associations of the lifestyle-based oxidative balance score with the number of cardiometabolic risk factors and cardiometabolic biomarkers by gender using linear regression.

	**SBP**	**DBP**	**FPG**	**TG**	**TC**	**HDLC**	**LDLC**	**Number of CMRFs**
**Male (*****n*** **= 277)**
LOBS^a^	– 0.438	0.057	– 0.061	– 0.117	– 0.046	0.034	– 0.022	– 0.076
95% CI	– 1.583, 0.708	– 0.658, 0.772	– 0.178, 0.057	– 0.194, – 0.039	– 0.104, 0.013	0.006, 0.061	– 0.076, 0.032	– 0.133, – 0.019
*P*-value	0.453	0.875	0.312	0.003	0.125	0.018	0.419	0.009
Tertile 1^b^	/	/	/	/	/	/	/	/
Tertile 2	– 3.923	– 1.367	– 0.023	– 0.297	– 0.189	0.070	– 0.145	– 0.303**
95% CI	– 8.361, 0.516	– 4.139, 1.404	– 0.483, 0.437	– 0.602, 0.008	– 0.417, 0.039	– 0.038, 0.179	– 0.354, 0.065	– 0.525, – 0.081
Tertile 3	– 3.608	– 3.248	– 0.117	– 0.378	– 0.062	0.189*	– 0.017	– 0.347*
95% CI	– 10.482, 3.266	– 7.541, 1.044	– 0.829, 0.595	– 0.851, 0.095	– 0.415, 0.292	0.021, 0.357	– 0.342, 0.307	– 0.691, – 0.003
P _trend_	0.099	0.107	0.768	0.032	0.282	0.021	0.447	0.005
**Female (*****n*** **= 433)**
LOBS ^a^	– 1.578	– 0.528	– 0.044	– 0.083	– 0.001	0.038	– 0.008	– 0.103
95% CI	– 3.030, – 0.126	– 1.373, 0.316	– 0.180, 0.092	– 0.161, – 0.005	– 0.082, 0.079	0.011, 0.065	– 0.077, 0.061	– 0.165, – 0.042
*P*– value	0.033	0.220	0.523	0.036	0.974	0.007	0.818	0.001
Tertile 1^b^	/	/	/	/	/	/	/	/
Tertile 2	– 3.011	– 2.505*	– 0.156	– 0.160	0.094	0.106**	0.094	– 0.177*
95% CI	– 7.145, 1.124	– 4.900, – 0.110	– 0.543, 0.232	– 0.380, 0.061	– 0.136, 0.323	0.028, 0.183	– 0.103, 0.291	– 0.352, – 0.002
Tertile 3	– 5.273	– 2.485	– 0.143	– 0.325*	0.048	0.162**	– 0.019	– 0.324**
95% CI	– 10.564, 0.020	– 5.550, 0.581	– 0.639, 0.353	– 0.608, – 0.042	– 0.246, 0.342	0.063, 0.261	– 0.271, 0.233	– 0.549, – 0.100
P _trend_	0.044	0.072	0.515	0.023	0.664	<0.001	0.967	0.004
P ${}_{\text{interaction}}^{\text{c}}$	0.322	0.473	0.841	0.534	0.269	0.664	0.634	0.617

SBP, systolic blood pressure; DBP, diastolic blood pressure; FPG, fasting plasma glucose; TG, triglycerides; TC, total cholesterol; HDLC, high-density lipoprotein cholesterol; LDLC, low-density lipoprotein cholesterol; CMRF, cardiometabolic risk factors; CI, Confidence interval.

^a^Regression coefficients and 95% CIs from multivariate linear regression models. Adjusted for age, educational degree, solitary status, and socio-economic degree of communities to which selected participants belonged.

^b^Grouped by tertile intervals, the first LOBS interval representing the preponderance of pro-oxidants was used as a reference. Tertile 1, LOBS−6~-1; Tertile 2, LOBS 0~1; Tertile 3, LOBS 2.

^c^P _interaction_ from stratified risk factor^*^LOBS interaction term in multivariate linear regression models. *, **, and *** respectively indicate *P* values < 0.05, < 0.01, and < 0.001.

**Table 5-2:** Associations of the lifestyle-based oxidative balance score with the number of cardiometabolic risk factors and cardiometabolic biomarkers by gender using logistic regression.

	**SBP^c^**	**DBP^c^**	**FPG^d^**	**TG^e^**	**TC^e^**	**HDLC^e^**	**LDLC^e^**	**Number of CMRFs**
**Male (*****n*** **= 277)**
LOBS ^a^	0.941	1.196	0.910	0.793	0.850	0.838	0.903	0.839
95% CI	0.815, 1.086	0.992, 1.442	0.749, 1.105	0.648, 0.970	0.632, 1.144	0.714, 0.983	0.650, 1.255	0.738, 0.954
*P*-value	0.406	0.061	0.342	0.024	0.284	0.030	0.545	0.007
Tertile 1^b^	/	/	/	/	/	/	/	/
Tertile 2	0.596	0.765	0.857	0.481	0.275	0.626	0.317	0.477**
95% CI	0.340, 1.044	0.365, 1.602	0.401, 1.832	0.206, 1.124	0.059, 1.277	0.333, 1.179	0.067, 1.511	0.288, 0.791
Tertile 3	0.626	1.350	0.671	0.779	0.421	0.418	0.520	0.466
95% CI	0.267, 1.466	0.479, 3.804	0.185, 2.429	0.245, 2.475	0.051, 3.481	0.135, 1.293	0.062, 4.374	0.217, 1.003
P _trend_	0.088	0.938	0.504	0.238	0.127	0.053	0.212	0.004
**Female (*****n*** **= 433)**
LOBS^a^	0.833	0.916	0.909	0.866	0.887	0.897	0.865	0.786
95% CI	0.707, 0.982	0.726, 1.154	0.722, 1.144	0.708, 1.060	0.718, 1.095	0.709, 1.133	0.674, 1.111	0.680, 0.907
*P*-value	0.029	0.456	0.414	0.163	0.264	0.361	0.255	0.001
Tertile 1^b^	/	/	/	/	/	/	/	/
Tertile 2	0.531**	0.632	1.029	0.845	1.099	0.636	0.978	0.656*
95% CI	0.334, 0.852	0.339, 1.178	0.546, 1.941	0.489, 1.461	0.610, 1.980	0.335, 1.206	0.493, 1.940	0.437, 0.984
Tertile 3	0.623	0.733	0.700	0.504	0.682	0.641	0.620	0.483**
95% CI	0.342, 1.136	0.325, 1.654	0.286, 1.713	0.227, 1.117	0.299, 1.558	0.274, 1.500	0.228, 1.684	0.287, 0.812
P _trend_	0.065	0.327	0.512	0.105	0.468	0.219	0.409	0.005
P ${}_{\text{interaction}}^{\text{f}}$	0.329	0.136	0.960	0.653	0.661	0.642	0.804	0.654

SBP, systolic blood pressure; DBP, diastolic blood pressure; FPG, fasting plasma glucose; TG, triglycerides; TC, total cholesterol; HDLC, high-density lipoprotein cholesterol; LDLC, low-density lipoprotein cholesterol; CMRF, cardiometabolic risk factors; CI, Confidence interval.

^a^Odds ratios and 95% CIs from multivariate logistic regression models. Adjusted for age, educational degree, solitary status, and socio-economic degree of communities to which selected participants belonged.

^b^Grouped by tertile intervals, the first LOBS interval representing the preponderance of pro-oxidants was used as a reference. Tertile 1, LOBS−6~-1; Tertile 2, LOBS 0~1; Tertile 3, LOBS 2.

^c^Blood pressure cutoffs: normal SBP, <140 mmHg, abnormal SBP, ≥140 mmHg; normal DBP, <90 mmHg, abnormal DBP, ≥90 mmHg.

^d^Fasting plasma glucose cutoffs: normal FPG, <7.0 mmol/L, abnormal FPG, ≥7.0 mmol/L.

^e^Lipids/lipoproteins cutoffs: normal TG, <2.3 mmol/L, abnormal TG, ≥2.3 mmol/L; normal TC, <6.2 mmol/L, abnormal TC, ≥6.2 mmol/L; normal HDLC, >1.02 mmol/L, abnormal HDLC, ≤ 1.0 mmol/L; normal LDLC, <4.1 mmol/L, abnormal LDLC, ≥4.1 mmol/L.

^f^P _interaction_ from stratified risk factor^*^LOBS interaction term in multivariate logistic regression models. *, **, and *** respectively indicate *P* values < 0.05, < 0.01, and < 0.001.

Participants were stratified based on the socioeconomic levels of their communities (Tables 6-1,6-2). The results of the linear regression model revealed that the inverse associations of LOBS (continuous variable) with LDLC and LOBS (interval) with DBP were only found among participants from communities with low socioeconomic levels. Moreover, the inverse linear association of LOBS (interval) with SBP was only found in individuals from communities with high socioeconomic levels. Whereas the associations of LOBS (continuous variable or interval) with TG and HDLC did not differ. In the logistic regression model, only the inverse associations of LOBS (interval) with abnormal SBP and LOBS (continuous variable) with abnormal TG were statistically significant in individuals from communities with high-level socioeconomic status. Furthermore, the inverse relationship between LOBS and the number of CMRFs did not differ by their socioeconomic levels.

**Table 6-1:** Associations of the lifestyle-based oxidative balance score with the number of cardiometabolic risk factors and cardiometabolic biomarkers by socio-economic degree using linear regression.

	**SBP**	**DBP**	**FPG**	**TG**	**TC**	**HDLC**	**LDLC**	**Number of CMRFs**
**Low level (*****n*** **= 268)**
LOBS^a^	– 0.733	– 0.594	– 0.038	– 0.080	– 0.083	0.038	– 0.083	– 0.092
95% CI	– 2.391, 0.925	– 1.572, 0.385	– 0.196, 0.120	– 0.158, – 0.015	– 0.168, 0.002	0.014, 0.062	– 0.155, – 0.010	– 0.161, – 0.022
*P*-value	0.385	0.233	0.637	0.046	0.055	0.002	0.026	0.010
Tertile 1^b^	/	/	/	/	/	/	/	/
Tertile 2	– 4.000	– 3.821*	– 0.005	– 0.234	– 0.107	0.100*	– 0.085	– 0.278*
95% CI	– 9.357, 1.358	– 6.963, – 0.680	– 0.518, 0.509	– 0.487, 0.019	– 0.384, 0.170	0.023, 0.178	– 0.323, 0.153	– 0.502, – 0.054
Tertile 3	– 4.371	– 4.210	– 0.065	– 0.376*	– 0.114	0.193***	– 0.199	– 0.436**
95% CI	– 11.844, 3.102	– 8.593, 0.173	– 0.781, 0.652	– 0.729, – 0.023	– 0.501, 0.273	0.085, 0.301	– 0.531, 0132	– 0.749, – 0.123
P _trend_	0.163	0.023	0.878	0.023	0.477	<0.001	0.233	0.003
**High level (*****n*** **= 442)**
LOBS ^a^	– 1.058	– 0.034	– 0.062	– 0.116	– 0.011	0.032	0.007	– 0.090
95% CI	– 2.171, 0.055	– 0.707, 0.638	– 0.170, 0.047	– 0.190, – 0.043	– 0.073, 0.051	0.004, 0.059	– 0.047, 0.062	– 0.142, – 0.037
*P*-value	0.062	0.920	0.265	0.002	0.730	0.023	0.789	<0.001
Tertile 1^b^	/	/	/	/	/	/	/	/
Tertile 2	– 2.984	– 1.153	– 0.164	– 0.218	– 0.022	0.089	0.003	– 0.207*
95% CI	– 6.647, 0.680	– 3.365, 1.059	– 0.522, 0.194	– 0.461, 0.025	– 0.227, 0.182	– 0.001, 0.179	– 0.178, 0184	– 0.380, – 0.034
Tertile 3	– 5.159*	– 1.675	– 0.132	– 0.355*	0.001	0.146*	0.009	– 0.297*
95% CI	– 10.081, – 0.238	– 4.647, 1.297	– 0.613, 0.350	– 0.681, – 0.029	– 0.274, 0.276	0.025, 0.267	– 0.234, 0.252	– 0.529, – 0.064
P _trend_	0.027	0.215	0.468	0.020	0.952	0.010	0.944	0.005
P _interaction_^c^	0.557	0.339	0.481	0.313	0.620	0.628	0.205	0.718

SBP, systolic blood pressure; DBP, diastolic blood pressure; FPG, fasting plasma glucose; TG, triglycerides; TC, total cholesterol; HDLC, high-density lipoprotein cholesterol; LDLC, low-density lipoprotein cholesterol; CMRF, cardiometabolic risk factors; CI, Confidence interval.

^a^Regression coefficients and 95% CIs from multivariate linear regression models. Adjusted for age, gender, educational degree, and solitary status.

^b^Grouped by tertile intervals, the first LOBS interval representing the preponderance of pro-oxidants was used as a reference. Tertile 1, LOBS−6~-1; Tertile 2, LOBS 0~1; Tertile 3, LOBS 2.

^c^P _interaction_ from stratified risk factor^*^LOBS interaction term in multivariate linear regression models. *, **, and *** respectively indicate *P* values < 0.05, < 0.01, and < 0.001.

**Table 6-2:** Associations of the lifestyle-based oxidative balance score with the number of cardiometabolic risk factors and cardiometabolic biomarkers by socio-economic degree using logistic regression.

	**SBP^c^**	**DBP^c^**	**FPG^d^**	**TG^e^**	**TC^e^**	**HDLC^e^**	**LDLC^e^**	**Number of CMRFs**
**Low level (*****n*** **= 268)**
LOBS^a^	0.900	1.010	0.889	0.884	0.770	0.854	– 0.037	0.818
95% CI	0.746, 1.087	0.815, 1.252	0.692, 1.141	0.691, 1.132	0.559, 1.059	0.688, 1.059	– 0.081, 0.007	0.697, 0.960
*P*-value	0.274	0.927	0.356	0.328	0.108	0.150	0.099	0.014
Tertile 1^b^	/	/	/	/	/	/	/	/
Tertile 2	0.479*	0.643	1.174	0.797	0.813	0.360**	0.815	0.529*
95% CI	0.256, 0.896	0.324, 1.275	0.531, 2.594	0.376, 1.689	0.339, 1.947	0.168, 0.773	0.298, 2.228	0.316, 0.887
Tertile 3	0.601	0.680	0.538	0.335	0.411	0.602	/	0.386**
95% CI	0.254, 1.426	0.254, 1.817	0.139, 2.086	0.088, 1.272	0.101, 1.671	0.216, 1.678	/	0.189, 0.786
P _trend_	0.104	0.285	0.568	0.124	0.234	0.072	0.073	0.004
**High level (*****n*** **= 442)**
LOBS ^a^	0.881	1.112	0.938	0.820	0.872	0.866	– 0.023	0.806
95% CI	0.771, 1.006	0.919, 1.345	0.778, 1.130	0.689, 0.974	0.707, 1.075	0.734, 1.022	−0.057, 0.010	0.714, 0.910
*P*-value	0.061	0.277	0.500	0.024	0.199	0.088	0.170	<0.001
Tertile 1^b^	/	/	/	/	/	/	/	/
Tertile 2	0.600*	0.739	0.827	0.699	0.828	0.853	0.704	0.600*
95% CI	0.387, 0.930	0.383, 1.428	0.448, 1.527	0.400, 1.222	0.431, 1.590	0.494, 1.475	0.330, 1.502	0.403, 0.892
Tertile 3	0.631	1.163	0.804	0.672	0.627	0.503	0.859	0.514*
95% CI	0.351, 1.134	0.514, 2.632	0.337, 1.918	0.313, 1.441	0.253, 1.554	0.209, 1.211	0.326, 2.260	0.302, 0.874
P _trend_	0.048	0.987	0.535	0.210	0.308	0.142	0.605	0.005
P _interaction_^f^	0.531	0.535	0.894	0.578	0.759	0.688	0.439	0.650

SBP, systolic blood pressure; DBP, diastolic blood pressure; FPG, fasting plasma glucose; TG, triglycerides; TC, total cholesterol; HDLC, high-density lipoprotein cholesterol; LDLC, low-density lipoprotein cholesterol; CMRF, cardiometabolic risk factors; CI, Confidence interval.

^a^Odds ratios and 95% CIs from multivariate logistic regression models. Adjusted for age, gender, educational degree, and solitary status.

^b^Grouped by tertile intervals, the first LOBS interval representing the preponderance of pro-oxidants was used as a reference. Tertile 1, LOBS−6~-1; Tertile 2, LOBS 0~1; Tertile 3, LOBS 2.

^c^Blood pressure cutoffs: normal SBP, <140 mmHg, abnormal SBP, ≥140 mmHg; normal DBP, <90 mmHg, abnormal DBP, ≥90 mmHg.

^d^Fasting plasma glucose cutoffs: normal FPG, <7.0 mmol/L, abnormal FPG, ≥7.0 mmol/L.

^e^Lipids/lipoproteins cutoffs: normal TG, <2.3 mmol/L, abnormal TG, ≥2.3 mmol/L; normal TC, <6.2 mmol/L, abnormal TC, ≥6.2 mmol/L; normal HDLC, >1.02 mmol/L, abnormal HDLC, ≤ 1.0 mmol/L; normal LDLC, <4.1 mmol/L, abnormal LDLC, ≥4.1 mmol/L.

^f^P _interaction_ from stratified risk factor^*^LOBS interaction term in multivariate logistic regression models. *, **, and *** respectively indicate *P* values < 0.05, < 0.01, and < 0.001.

### Sensitivity analysis

For sensitivity analysis, new LOBS scores were calculated by separately removing each component of LOBS. The associations of LOBS with abnormal SBP, TG, and HDLC remained unchanged regardless of physical activity, smoking, or alcohol consumption removal. The LOBS that did not contain physical activity component showed a significant inverse association with abnormal FPG. Notably, the LOBS without the BMI component was significantly associated with cardiometabolic biomarkers, including inverse associations with TG, TC, and LDLC in linear regression models, and inverse associations with abnormal DBP, TC, and LDLC in logistic regression models. In both linear and logistic models, all LOBS with three components had a significant inverse association with the number of CMRFs (all *P* < 0.05). Moreover, the relationships between the LOBS quartile intervals and cardiometabolic biomarkers as well as the number of CMRFs were consistent with those in the primary analysis. Detailed results are available in the Supplemental materials.

## Discussion

In this work, we examined the associations of LOBS based on a priori methods with cardiometabolic biomarkers and CMRF numbers. Poor lifestyle patterns were linked to a higher cardiometabolic risk. Long-term cellular damage as a result of oxidative stress (OS) during aging accelerates the accumulation of CMRFs, including hypertension, diabetes, and dyslipidemia (21, 22), causing fatal cardiovascular events (23, 24). Studies have shown that the complex roles of exogenous modifiable factors and endogenous mechanisms confound the independent effects of pro-oxidants or antioxidants on health outcomes. Oxidative balance score or oxidative stress score (13) are used for the comprehensive measurement of pro-oxidant and antioxidant exposure. Based on a previous review of oxidative balance scores summarized by Hernandez-Ruiz et al. (15), and the database availability of our original cross-sectional study, we selected the components of physical activity, smoking status, alcohol consumption, and BMI level to construct the LOBS. In addition to diet and sleep, these components cover all conventional lifestyle factors. Considering the obesity characteristics of the Chinese elderly population, the cut-off values of BMI levels were adjusted. Unlike in the report by Mao et al. (25), overweight (24.00–27.99 kg/m^2^) and obesity (>28.00 kg/m^2^) were used as different levels of pro-oxidant exposure. In all models, participants had few concurrent CMRFs with higher LOBS scores, indicating more dominant lifestyle exposure to antioxidants. The odds of co-existing CMRFs decreased by about 54% if participants were at the highest antioxidant level for all lifestyles, which has significant preventative and therapeutic implications.

We found a significant inverse association between LOBS and SBP among participants of older age (>70 years), women, and those from communities with high socioeconomic levels. Previous studies revealed that SBP appears to linearly increase with age and is more evident in females, particularly postmenopausal women (26–28). The intercept of SBP would be higher in the presence of other risk factors (29). Therefore, a healthier lifestyle depicted by a better LOBS would help manage the SBP. Also, a decrease in NAD+-dependent deacetylase activity of the metabolic sensor *Sirt3* with aging is accompanied by a decrease in mitochondrial energy metabolism and an increase in the production of reactive oxygen species (ROS) (30). Excess ROS enhances the inflexibility of the large arteries and disrupts their mechanical properties and compliance, resulting in significantly increased resistance to blood flow and thus increased SBP (31). This suggests that the antioxidant effect of an exogenous lifestyle should be investigated because endogenous oxidative stress regulatory mechanisms gradually lose their function. In addition, a reverse association between LOBS intervals and DBP was noted only in participants from the lower age group and communities with low socioeconomic levels, however, we detected no association of LOBS with abnormal DBP.

Due to the limited number of participants with diabetes, we did not detect a significant association between LOBS and FPG. An inverse association between LOBS and abnormal FPG was only observed in the upper age group; nevertheless, cross-interval comparisons revealed no significant dose-response relationship. Additional sensitivity analysis outcomes revealed a statistically significant inverse relationship between LOBS excluding physical activity and FPG, whereas no such trend was noted in the cross-interval comparisons. This may be attributed to a failure to collect data regarding antidiabetic use, which underestimates the true effect of LOBS on glycemic control.

Furthermore, an inverse association of LOBS with TG and a positive association with HDLC were noted in nearly all models. Notably, increased oxidative stress is simultaneously linked to high TG and low HDLC (32, 33). Plasma lipid peroxidation accompanied by high oxidative stress causes insulin resistance (34), consequently increasing the TG levels (35). On the other hand, hypertriglyceridemia induces oxidative stress that triggers CMDs (36), with increased plasma lipid peroxidation and low levels of HDLC (37). In this study, the relationship between LOBS and HDLC corresponded to the hypothesis which suggests that LOBS causes oxidative stress. As reported by Zelzer et al. (38), HDLC levels can reflect the effects of oxidative stress on lipid metabolism, independent of age and sex. In the present study, the association of LOBS with HDLC similarly did not differ by age and sex.

Previous studies on LOBS focused on the relationship between oxidative status and the risk of chronic diseases. Few studies have examined oxidative stress and cardiometabolic health among the community-dwelling elderly. This is cardiometabolic health research on the community-dwelling elderly population performed in the multicenter setting. The LOBS constructed provides a measure of the combined effects of pro- and anti-oxidant lifestyle factors that predicts cardiometabolic health by exogenous agents. Additionally, we established a priori cutoff values for LOBS, hence effectively minimizing the subjectivity of measurements. We found a significant inverse association between LOBS and the number of cardiometabolic risk factors, which provides important guidance for the healthy lifestyle management of older adults in community-based general practice.

One shortcoming of this work is that diet and sleep quality may influence oxidative stress. Due to the lack of data from our previous cross-sectional studies, they could not be taken into consideration and discussion. In the future, we will use validated questionnaires in further cohort studies in order to assess their anti-oxidant and pro-oxidant effects. Secondly, the smoking status (packs/year) of smokers or former smokers could not be collected in this analysis. As a major reason, in previous studies the OBS, constructed by a priori methods, rarely weighted the smoking component quantitatively by pack/year, making it difficult for us to measure its weight accurately. Third, physical activity levels depended on self-reports rather than validated questionnaire scales (e.g., the International Physical Activity Scale IPAQ), which may lead to misclassification of participants' physical activity levels. However, after excluding the physical activity component from the LOBS, results in the sensitivity analysis were still consistent with the primary analysis. Fourth, missing data on medication use (antihypertensive, antidiabetics, statins) led to an underestimation of CMRF numbers. Nevertheless, the LOBS had a significant negative relationship with the number of CMRFs in all models. Eventually, the cross-sectional design of the original study precluded the possibility of a causal explanation and the sample was obtained from a higher economic level region of a middle-income country. This may limit the extrapolation of our findings beyond the studied population. Therefore, a larger sample size with more varied regional sources is necessary to validate our findings.

## Conclusion

In conclusion, we found that ideal LOBS significantly reduces the number of cardiometabolic risk factors in community-dwelling elderly people, regardless of their age, gender, and socio-economic level. Therefore, comprehensive healthy lifestyle management can improve cardiometabolic health in older adults, and hence should be implemented by general practitioners at the community level.

## Data availability statement

The raw data supporting the conclusions of this article will be made available by the authors, without undue reservation.

## Ethics statement

The studies involving human participants were reviewed and approved by Ethics Committee of Shanghai East Hospital, Tongji University School of Medicine. The patients/participants provided their written informed consent to participate in this study.

## Author contributions

YL: conceptualization, methodology, formal analysis, and writing—original draft. HY: validation and writing—review and editing. QL: data curation and writing—review editing. SG, XC, and YZ: investigation. HJ: funding acquisition, supervision, project administration, and writing—review and editing. All authors contributed to the article and approved the submitted version.

## Funding

This study was supported by Municipal Health Commission of Pudong New Area (PW2019E-4) and Shanghai Municipal Health Commission (202140248).

## Conflict of interest

The authors declare that the research was conducted in the absence of any commercial or financial relationships that could be construed as a potential conflict of interest.

## Publisher's note

All claims expressed in this article are solely those of the authors and do not necessarily represent those of their affiliated organizations, or those of the publisher, the editors and the reviewers. Any product that may be evaluated in this article, or claim that may be made by its manufacturer, is not guaranteed or endorsed by the publisher.
