# Supplementary material for: Lifestyle-based oxidative balance score and its association with cardiometabolic health of the community-dwelling elderly: A cross-sectional secondary analysis
Source: Front Cardiovasc Med. 2022 Sep 27;9:1000546. doi: 10.3389/fcvm.2022.1000546 (PMC9551053; doi:10.3389/fcvm.2022.1000546)
Supplement: Supplementary file 1 [file Data_Sheet_1.docx]

**Table S1. Correlation coefficient and** **significance test matrix of LOBS with white blood cell count (WBC) and neutrophil percentage (NEUT%) by Spearman's rank correlation analysis.**

|  | LOBS | WBC | NEUT |
| --- | --- | --- | --- |
| LOBS | 1.0000 |  |  |
| WBC | -0.1445  <0.001 | 1.0000 |  |
| NEUT | 0.0138  0.7147 | 0.2643  <0.001 | 1.0000 |

**Table S2-1. Associations of LOBS (****except physical activity) with the number of CMRFs and cardiometabolic biomarkers using linear regression.**

|  | **SBP** | **DBP** | **FPG** | **TG** | **TC** | **HDLC** | **LDLC** | **Number of CMRFs** |
| --- | --- | --- | --- | --- | --- | --- | --- | --- |
| LOBS ^a^ | -2.229 | -0.736 | -0.122 | -0.117 | 0.006 | 0.055 | 0.001 | -0.123 |
| 95%CI | -3.538, -0.919 | -1.524, 0.052 | -0.249, 0.006 | -0.194, -0.040 | -0.065, 0.078 | 0.027, 0.082 | -0.062, 0.063 | -0.183, -0.064 |
| P-value | <0.001 | 0.067 | 0.061 | 0.003 | 0.862 | <0.001 | 0.978 | <0.001 |
| Physical Activity |  |  |  |  |  |  |  |  |
| Low ^b^ | / | / | / | / | / | / | / | / |
| Moderate ^b^ | 3.494 | -0.193 | -0.031 | -0.025 | 0.087 | -0.007 | 0.164 | -0.061 |
| 95%CI | -0.829, 7.817 | -2.794, 2.408 | -0.451, 0.389 | -0.280, 0.230 | -0.149, 0.323 | -0.098, 0.084 | -0.042, 0.371 | -0.256, 0.134 |
| P-value | 0.113 | 0.884 | 0.885 | 0.850 | 0.468 | 0.879 | 0.119 | 0.539 |
| High ^b^ | 0.964 | 0.676 | 0.059 | -0.158 | -0.143 | 0.019 | -0.083 | -0.098 |
| 95%CI | -1.907, 3.835 | -1.052, 2.403 | -0.220, 0.338 | -0.327, 0.011 | -0.300, 0.013 | -0.041, 0.079 | -0.220, 0.054 | -0.228, 0.031 |
| P-value | 0.510 | 0.443 | 0.680 | 0.067 | 0.073 | 0.534 | 0.237 | 0.137 |

Abbreviations: SBP, systolic blood pressure; DBP, diastolic blood pressure; FPG, fasting plasma glucose; TG, triglycerides; TC, total cholesterol; HDLC, high-density lipoprotein cholesterol; LDLC, low-density lipoprotein cholesterol; CMRF, cardiometabolic risk factors; CI, Confidence interval.

a. Regression coefficients and 95% CIs from multivariate linear regression models. Adjusted for physical activity, age, gender, educational degree, solitary status, and socio-economic degree of communities to which selected participants belonged.

b. Grouped by physical activity, participants with low physical activity levels were used as a reference. Adjusted for LOBS (except physical activity), age, gender, educational degree, solitary status, and socio-economic degree of communities to which selected participants belonged.

**Table S2-2. Associations of LOBS (except physical activity) with the number of CMRFs and cardiometabolic biomarkers using logistic regression.**

|  | **SBP ^c^** | **DBP ^c^** | **FPG ^d^** | **TG ^e^** | **TC ^e^** | **HDLC ^e^** | **LDLC ^e^** | **Number of CMRFs** |
| --- | --- | --- | --- | --- | --- | --- | --- | --- |
| LOBS ^a^ | 0.781 | 0.938 | 0.791 | 0.880 | 1.091 | 0.828 | 1.155 | 0.765 |
| 95%CI | 0.666, 0.916 | 0.772, 1.140 | 0.644, 0.972 | 0.722, 1.073 | 0.841, 1.416 | 0.693, 0.990 | 0.852, 1.565 | 0.666, 0.878 |
| P-value | 0.002 | 0.522 | 0.025 | 0.207 | 0.513 | 0.038 | 0.354 | <0.001 |
| Physical Activity |  |  |  |  |  |  |  |  |
| Low ^b^ | / | / | / | / | / | / | / | / |
| Moderate ^b^ | 1.287 | 1.304 | 0.971 | 0.958 | 0.870 | 0.584 | 0.847 | 0.812 |
| 95%CI | 0.768, 2.157 | 0.680, 2.498 | 0.480, 1.965 | 0.520, 1.765 | 0.429, 1.768 | 0.287, 1.188 | 0.369, 1.944 | 0.510, 1.293 |
| P-value | 0.338 | 0.424 | 0.935 | 0.890 | 0.701 | 0.138 | 0.695 | 0.380 |
| High ^b^ | 1.061 | 1.547 | 1.162 | 0.628 | 0.459 | 0.829 | 0.437 | 0.755 |
| 95%CI | 0.758, 1.485 | 0.988, 2.423 | 0.729, 1.853 | 0.401, 0.984 | 0.270, 0.781 | 0.540, 1.273 | 0.232, 0.821 | 0.561, 1.017 |
| P-value | 0.729 | 0.057 | 0.527 | 0.042 | 0.004 | 0.391 | 0.010 | 0.065 |

Abbreviations: SBP: systolic blood pressure, DBP: diastolic blood pressure, FPG: fasting plasma glucose, TG: triglycerides, TC: total cholesterol, HDLC: high-density lipoprotein cholesterol, LDLC: low-density lipoprotein cholesterol, CMRF: cardiometabolic risk factors; CI, Confidence interval.

a. Odds ratios and 95% CIs from multivariate logistic regression models. Adjusted for physical activity, age, gender, educational degree, solitary status, and socio-economic degree of communities to which selected participants belonged.

b. Grouped by physical activity, participants with low physical activity levels were used as a reference. Adjusted for LOBS (except physical activity), age, gender, educational degree, solitary status, and socio-economic degree of communities to which selected participants belonged.

c. Blood pressure cutoffs: normal SBP, <140mmHg, abnormal SBP, ≥140 mmHg; normal DBP, <90 mmHg, abnormal DBP, ≥90 mmHg.

d. Fasting plasma glucose cutoffs: normal FPG, <7.0 mmol/L, abnormal FPG, ≥7.0 mmol/L.

e. Lipids/lipoproteins cutoffs: normal TG, <2.3mmol/L, abnormal TG, ≥2.3mmol/L; normal TC, <6.2mmol/L, abnormal TC, ≥6.2mmol/L; normal HDLC, >1.02mmol/L, abnormal HDLC, 1.0≤mmol/L; normal LDLC, <4.1mmol/L, abnormal LDLC, ≥4.1mmol/L.

**Table S3-1. Associations of LOBS (except smoking) with the number of CMRFs and cardiometabolic biomarkers using linear regression.**

|  | **SBP** | **DBP** | **FPG** | **TG** | **TC** | **HDLC** | **LDLC** | **Number of CMRFs** |
| --- | --- | --- | --- | --- | --- | --- | --- | --- |
| LOBS ^a^ | -1.118 | -0.399 | -0.032 | -0.128 | -0.021 | 0.033 | 0.005 | -0.095 |
| 95%CI | -2.186, -0.050 | -1.040, 0.241 | -0.136, 0.071 | -0.190, -0.065 | -0.079, 0.037 | 0.011, 0.055 | -0.046, 0.056 | -0.142, -0.047 |
| P-value | 0.040 | 0.221 | 0.540 | <0.001 | 0.480 | 0.004 | 0.850 | <0.001 |
| Smoking |  |  |  |  |  |  |  |  |
| Never ^b^ | / | / | / | / | / | / | / | / |
| Former ^b^ | 4.001 | 0.167 | 0.307 | -0.086 | 0.058 | -0.113 | 0.220 | 0.252 |
| 95%CI | -1.684, 9.687 | -3.243, 3.577 | -0.244, 0.857 | -0.420, 0.247 | -0.252, 0.368 | -0.232, 0.006 | -0.050, 0.491 | -0.003, 0.507 |
| P-value | 0.168 | 0.924 | 0.274 | 0.611 | 0.713 | 0.062 | 0.110 | 0.052 |
| Current ^b^ | -0.826 | -1.245 | 0.202 | -0.017 | 0.148 | -0.041 | 0.210 | 0.040 |
| 95%CI | -6.007, 4.355 | -4.352, 1.862 | -0.300, 0.703 | -0.321, 0.286 | -0.133, 0.431 | -0.149, 0.068 | -0.036, 0.457 | -0.193, 0.272 |
| P-value | 0.754 | 0.432 | 0.430 | 0.910 | 0.301 | 0.461 | 0.094 | 0.738 |

Abbreviations: SBP, systolic blood pressure; DBP, diastolic blood pressure; FPG, fasting plasma glucose; TG, triglycerides; TC, total cholesterol; HDLC, high-density lipoprotein cholesterol; LDLC, low-density lipoprotein cholesterol; CMRF, cardiometabolic risk factors; CI, Confidence interval.

a. Regression coefficients and 95% CIs from multivariate linear regression models. Adjusted for smoking, age, gender, educational degree, solitary status, and socio-economic degree of communities to which selected participants belonged.

b. Grouped by smoking status, participants who had never smoked were used as a reference. Adjusted for LOBS (except smoking), age, gender, educational degree, solitary status, and socio-economic degree of communities to which selected participants belonged.

**Table S3-2. Associations of LOBS (except smoking) with the number of CMRFs and cardiometabolic biomarkers using logistic regression.**

|  | **SBP ^c^** | **DBP ^c^** | **FPG ^d^** | **TG ^e^** | **TC ^e^** | **HDLC ^e^** | **LDLC ^e^** | **Number of CMRFs** |
| --- | --- | --- | --- | --- | --- | --- | --- | --- |
| LOBS ^a^ | 0.870 | 1.070 | 0.984 | 0.790 | 0.873 | 0.824 | 0.919 | 0.798 |
| 95%CI | 0.768, 0.986 | 0.905, 1.264 | 0.828, 1.171 | 0.672, 0.930 | 0.724, 1.052 | 0.703, 0.966 | 0.740, 1.143 | 0.714, 0.891 |
| P-value | 0.030 | 0.430 | 0.858 | 0.005 | 0.154 | 0.017 | 0.448 | <0.001 |
| Smoking |  |  |  |  |  |  |  |  |
| Never ^b^ | / | / | / | / | / | / | / | / |
| Former ^b^ | 1.118 | 1.129 | 2.087 | 0.937 | 1.244 | 1.705 | 1.848 | 1.679 |
| 95%CI | 0.575, 2.172 | 0.496, 2.572 | 0.897, 4.854 | 0.396, 2.217 | 0.359, 4.306 | 0.857, 3.392 | 0.498, 6.862 | 0.931, 3.025 |
| P-value | 0.743 | 0.772 | 0.088 | 0.883 | 0.731 | 0.128 | 0.359 | 0.085 |
| Current ^b^ | 1.009 | 0.871 | 2.076 | 0.765 | 1.572 | 0.740 | 2.012 | 1.103 |
| 95%CI | 0.551, 1.845 | 0.402, 1.888 | 0.940, 4.589 | 0.330, 1.773 | 0.534, 4.626 | 0.362, 1.512 | 0.602, 6.722 | 0.650, 1.873 |
| P-value | 0.978 | 0.727 | 0.071 | 0.532 | 0.412 | 0.409 | 0.256 | 0.717 |

Abbreviations: SBP: systolic blood pressure, DBP: diastolic blood pressure, FPG: fasting plasma glucose, TG: triglycerides, TC: total cholesterol, HDLC: high-density lipoprotein cholesterol, LDLC: low-density lipoprotein cholesterol, CMRF: cardiometabolic risk factors; CI, Confidence interval.

a. Odds ratios and 95% CIs from multivariate logistic regression models. Adjusted for smoking, age, gender, educational degree, solitary status, and socio-economic degree of communities to which selected participants belonged.

b. Grouped by smoking status, participants who had never smoked were used as a reference. Adjusted for LOBS (except smoking), age, gender, educational degree, solitary status, and socio-economic degree of communities to which selected participants belonged.

c. Blood pressure cutoffs: normal SBP, <140mmHg, abnormal SBP, ≥140 mmHg; normal DBP, <90 mmHg, abnormal DBP, ≥90 mmHg.

d. Fasting plasma glucose cutoffs: normal FPG, <7.0 mmol/L, abnormal FPG, ≥7.0 mmol/L.

e. Lipids/lipoproteins cutoffs: normal TG, <2.3mmol/L, abnormal TG, ≥2.3mmol/L; normal TC, <6.2mmol/L, abnormal TC, ≥6.2mmol/L; normal HDLC, >1.02mmol/L, abnormal HDLC, 1.0≤mmol/L; normal LDLC, <4.1mmol/L, abnormal LDLC, ≥4.1mmol/L.

**Table S4-1. Associations of LOBS (except alcohol consumption) with the number of CMRFs and cardiometabolic biomarkers using linear regression.**

|  | **SBP** | **DBP** | **FPG** | **TG** | **TC** | **HDLC** | **LDLC** | **Number of CMRFs** |
| --- | --- | --- | --- | --- | --- | --- | --- | --- |
| LOBS ^a^ | -1.002 | -0.353 | -0.048 | -0.101 | -0.017 | 0.041 | -0.010 | -0.099 |
| 95%CI | -1.997, -0.008 | -0.949, 0.242 | -0.144, 0.048 | -0.159, -0.043 | -0.071, 0.037 | 0.020, 0.062 | -0.057, 0.038 | -0.143, -0.054 |
| P-value | 0.048 | 0.245 | 0.329 | <0.001 | 0.532 | <0.001 | 0.690 | <0.001 |
| Alcohol Consumption |  |  |  |  |  |  |  |  |
| Never ^b^ | / | / | / | / | / | / | / | / |
| Moderate ^b^ | -1.081 | -0.645 | 0.252 | -0.056 | -0.004 | 0.056 | -0.005 | 0.005 |
| 95%CI | -6.579, 4.416 | -3.937, 2.647 | -0.279, 0.784 | -0.378, 0.266 | -0.302, 0.295 | -0.059, 0.170 | -0.266, 0.257 | -0.241, 0.252 |
| P-value | 0.700 | 0.701 | 0.352 | 0.733 | 0.981 | 0.341 | 0.971 | 0.967 |
| Heavy ^b^ | 3.288 | -1.641 | 0.077 | 0.282 | 0.397 | 0.043 | 0.290 | 0.011 |
| 95%CI | -5.120,11.695 | -6.676, 3.394 | -0.736, 0.890 | -0.211, 0.774 | -0.060, 0.853 | -0.133, 0.218 | -0.110, 0.690 | -0.366, 0.388 |
| P-value | 0.443 | 0.523 | 0.852 | 0.262 | 0.088 | 0.634 | 0.155 | 0.953 |

Abbreviations: SBP, systolic blood pressure; DBP, diastolic blood pressure; FPG, fasting plasma glucose; TG, triglycerides; TC, total cholesterol; HDLC, high-density lipoprotein cholesterol; LDLC, low-density lipoprotein cholesterol; CMRF, cardiometabolic risk factors; CI, Confidence interval.

a. Regression coefficients and 95% CIs from multivariate linear regression models. Adjusted for alcohol consumption, age, gender, educational degree, solitary status, and socio-economic degree of communities to which selected participants belonged.

b. Grouped by alcohol consumption, participants who had never drunk were used as a reference. Adjusted for LOBS (except alcohol consumption), age, gender, educational degree, solitary status, and socio-economic degree of communities to which selected participants belonged.

**Table S4-2. Associations of LOBS (except alcohol consumption) with the number of CMRFs and cardiometabolic biomarkers using logistic regression.**

|  | **SBP ^c^** | **DBP ^c^** | **FPG ^d^** | **TG ^e^** | **TC ^e^** | **HDLC ^e^** | **LDLC ^e^** | **Number of CMRFs** |
| --- | --- | --- | --- | --- | --- | --- | --- | --- |
| LOBS ^a^ | 0.875 | 1.009 | 0.905 | 0.849 | 0.857 | 0.818 | 0.854 | 0.796 |
| 95%CI | 0.779, 0.983 | 0.866, 1.186 | 0.771, 1.062 | 0.730, 0.988 | 0.717, 1.025 | 0.707, 0.945 | 0.695, 1.049 | 0.718, 0.881 |
| P-value | 0.025 | 0.911 | 0.222 | 0.034 | 0.091 | 0.007 | 0.131 | <0.001 |
| Alcohol Consumption |  |  |  |  |  |  |  |  |
| Never ^b^ | / | / | / | / | / | / | / | / |
| Moderate ^b^ | 0.927 | 0.467 | 1.378 | 0.979 | 1.552 | 0.707 | 1.486 | 1.071 |
| 95%CI | 0.492, 1.744 | 0.175, 1.247 | 0.612, 3.100 | 0.407, 2.356 | 0.547, 4.400 | 0.328, 1.525 | 0.472, 4.674 | 0.601, 1.908 |
| P-value | 0.814 | 0.129 | 0.439 | 0.963 | 0.409 | 0.376 | 0.498 | 0.815 |
| Heavy ^b^ | 0.994 | 0.370 | 0.989 | 1.947 | 0.804 | 0.702 | / | 0.981 |
| 95%CI | 0.363, 2.724 | 0.080, 1.699 | 0.266, 3.674 | 0.639, 5.929 | 0.098, 6.607 | 0.236, 2.084 | / | 0.407, 2.365 |
| P-value | 0.991 | 0.201 | 0.987 | 0.241 | 0.839 | 0.523 | / | 0.967 |

Abbreviations: SBP: systolic blood pressure, DBP: diastolic blood pressure, FPG: fasting plasma glucose, TG: triglycerides, TC: total cholesterol, HDLC: high-density lipoprotein cholesterol, LDLC: low-density lipoprotein cholesterol, CMRF: cardiometabolic risk factors; CI, Confidence interval.

a. Odds ratios and 95% CIs from multivariate logistic regression models. Adjusted for alcohol consumption, age, gender, educational degree, solitary status, and socio-economic degree of communities to which selected participants belonged.

b. Grouped by alcohol consumption, participants who had never drunk were used as a reference. Adjusted for LOBS (except alcohol consumption), age, gender, educational degree, solitary status, and socio-economic degree of communities to which selected participants belonged.

c. Blood pressure cutoffs: normal SBP, <140mmHg, abnormal SBP, ≥140 mmHg; normal DBP, <90 mmHg, abnormal DBP, ≥90 mmHg.

d. Fasting plasma glucose cutoffs: normal FPG, <7.0 mmol/L, abnormal FPG, ≥7.0 mmol/L.

e. Lipids/lipoproteins cutoffs: normal TG, <2.3mmol/L, abnormal TG, ≥2.3mmol/L; normal TC, <6.2mmol/L, abnormal TC, ≥6.2mmol/L; normal HDLC, >1.02mmol/L, abnormal HDLC, 1.0≤mmol/L; normal LDLC, <4.1mmol/L, abnormal LDLC, ≥4.1mmol/L.

**Table S5-1. Associations of LOBS (except overweight/obesity) with the number of CMRFs and cardiometabolic biomarkers using linear regression.**

|  | **SBP** | **DBP** | **FPG** | **TG** | **TC** | **HDLC** | **LDLC** | **Number of CMRFs** |
| --- | --- | --- | --- | --- | --- | --- | --- | --- |
| LOBS ^a^ | 0.104 | 0.330 | -0.024 | -0.064 | -0.070 | 0.012 | -0.056 | -0.049 |
| 95%CI | -0.978, 1.186 | -0.319, 0.978 | -0.129, 0.082 | -0.128, -0.0002 | -0.129, -0.011 | -0.010, 0.035 | -0.107, -0.004 | -0.097,-0.0002 |
| P-value | 0.850 | 0.318 | 0.657 | 0.049 | 0.020 | 0.292 | 0.035 | 0.0499 |
| BMI level |  |  |  |  |  |  |  |  |
| Underweight/  Normal ^b^ | / | / | / | / | / | / | / | / |
| Overweight ^b^ | 4.628 | 3.067 | 0.100 | 0.306 | -0.190 | -0.131 | -0.162 | 0.259 |
| 95%CI | 1.731, 7.524 | 1.332, 4.803 | -0.183, 0.382 | 0.135, 0.476 | -0.348, -0.032 | -0.191, -0.070 | -0.300, -0.023 | 0.129, 0.389 |
| P-value | 0.002 | <0.001 | 0.489 | <0.001 | 0.019 | <0.001 | 0.022 | <0.001 |
| Obesity ^b^ | 8.022 | 3.168 | 0.280 | 0.356 | -0.117 | -0.177 | -0.131 | 0.384 |
| 95%CI | 4.038, 12.006 | 0.782, 5.555 | -0.108, 0.669 | 0.122, 0.591 | -0.334, 0.101 | -0.260, -0.094 | -0.321, 0.059 | 0.205, 0.564 |
| P-value | <0.001 | 0.009 | 0.157 | 0.003 | 0.292 | <0.001 | 0.176 | <0.001 |

Abbreviations: SBP, systolic blood pressure; DBP, diastolic blood pressure; FPG, fasting plasma glucose; TG, triglycerides; TC, total cholesterol; HDLC, high-density lipoprotein cholesterol; LDLC, low-density lipoprotein cholesterol; CMRF, cardiometabolic risk factors; CI, Confidence interval.

a. Regression coefficients and 95% CIs from multivariate linear regression models. Adjusted for BMI level, age, gender, educational degree, solitary status, and socio-economic degree of communities to which selected participants belonged.

b. Grouped by BMI level, participants with underweight or normal weight were used as a reference. Adjusted for LOBS (except overweight/obesity), age, gender, educational degree, solitary status, and socio-economic degree of communities to which selected participants belonged.

**Table S5-2. Associations of LOBS (except overweight/obesity) with the number of CMRFs and cardiometabolic biomarkers using logistic regression.**

|  | **SBP ^c^** | **DBP ^c^** | **FPG ^d^** | **TG ^e^** | **TC ^e^** | **HDLC ^e^** | **LDLC ^e^** | **Number of CMRFs** |
| --- | --- | --- | --- | --- | --- | --- | --- | --- |
| LOBS ^a^ | 1.003 | 1.218 | 0.937 | 0.853 | 0.733 | 0.967 | 0.741 | 0.884 |
| 95%CI | 0.882, 1.139 | 1.027, 1.446 | 0.787, 1.115 | 0.722, 1.008 | 0.596, 0.903 | 0.829, 1.127 | 0.585, 0.939 | 0.788, 0.992 |
| P-value | 0.969 | 0.024 | 0.464 | 0.061 | 0.003 | 0.664 | 0.013 | 0.035 |
| BMI level |  |  |  |  |  |  |  |  |
| Underweight/  Normal ^b^ | / | / | / | / | / | / | / | / |
| Overweight ^b^ | 1.658 | 1.773 | 1.335 | 1.457 | 0.896 | 2.146 | 0.547 | 1.759 |
| 95%CI | 1.179, 2.331 | 1.108, 2.836 | 0.826, 2.158 | 0.934, 2.272 | 0.545, 1.472 | 1.348, 3.417 | 0.299, 1.000 | 1.298, 2.384 |
| P-value | 0.004 | 0.017 | 0.239 | 0.097 | 0.664 | 0.001 | 0.04995 | <0.001 |
| Obesity ^b^ | 2.521 | 1.819 | 1.422 | 1.463 | 0.567 | 2.727 | 0.639 | 2.305 |
| 95%CI | 1.544, 4.115 | 0.975, 3.394 | 0.756, 2.675 | 0.811, 2.641 | 0.266, 1.207 | 1.516, 4.906 | 0.288, 1.422 | 1.515, 3.506 |
| P-value | <0.001 | 0.060 | 0.275 | 0.206 | 0.141 | <0.001 | 0.273 | <0.001 |

Abbreviations: SBP: systolic blood pressure, DBP: diastolic blood pressure, FPG: fasting plasma glucose, TG: triglycerides, TC: total cholesterol, HDLC: high-density lipoprotein cholesterol, LDLC: low-density lipoprotein cholesterol, CMRF: cardiometabolic risk factors; CI, Confidence interval.

a. Odds ratios and 95% CIs from multivariate logistic regression models. Adjusted for BMI level, age, gender, educational degree, solitary status, and socio-economic degree of communities to which selected participants belonged.

b. Grouped by BMI level, participants with underweight or normal weight were used as a reference. Adjusted for LOBS (except overweight/obesity), age, gender, educational degree, solitary status, and socio-economic degree of communities to which selected participants belonged.

c. Blood pressure cutoffs: normal SBP, <140mmHg, abnormal SBP, ≥140 mmHg; normal DBP, <90 mmHg, abnormal DBP, ≥90 mmHg.

d. Fasting plasma glucose cutoffs: normal FPG, <7.0 mmol/L, abnormal FPG, ≥7.0 mmol/L.

e. Lipids/lipoproteins cutoffs: normal TG, <2.3mmol/L, abnormal TG, ≥2.3mmol/L; normal TC, <6.2mmol/L, abnormal TC, ≥6.2mmol/L; normal HDLC, >1.02mmol/L, abnormal HDLC, 1.0≤mmol/L; normal LDLC, <4.1mmol/L, abnormal LDLC, ≥4.1mmol/L.

**Table S6-1. Associations of the lifestyle-based oxidative balance score (quartile intervals) with the number of CMRFs and cardiometabolic biomarkers using linear regression.**

|  | **SBP** | **DBP** | **FPG** | **TG** | **TC** | **HDLC** | **LDLC** | **Number of CMRFs** |
| --- | --- | --- | --- | --- | --- | --- | --- | --- |
| Quartile 1^b^ | / | / | / | / | / | / | / | / |
| Quartile 2 | -4.287* | -3.677*** | -0.065 | -0.195 | 0.037 | 0.121** | 0.008 | -0.198* |
| 95%CI | -7.749, -0.825 | -5.734, -1.619 | -0.400, 0.271 | -0.399, 0.009 | -0.152, 0.225 | 0.049, 0.193 | -0.158, 0.173 | -0.354, -0.042 |
| Quartile 3 | -2.437 | 0.053 | -0.143 | -0.239* | -0.114 | 0.055 | -0.021 | -0.273** |
| 95%CI | -6.260, 1.385 | -2.218, 2.325 | -0.514, 0.228 | -0.465, -0.014 | -0.322, 0.094 | -0.025, 0.134 | -0.204, 0.161 | -0.445, -0.101 |
| Quartile 4 | -5.124* | -2.626* | -0.110 | -0.3556** | -0.024 | 0.163*** | -0.052 | -0.346*** |
| 95%CI | -9.228, -1.020 | -5.064, -0.187 | -0.508, 0.288 | -0.598, -0.115 | -0.248, 0.199 | 0.078, 0.249 | -0.248, 0.144 | -0.531, -0.161 |
| P _trend_ | 0.022 | 0.179 | 0.464 | 0.002 | 0.498 | 0.001 | 0.594 | <0.001 |

* P<0.05, ** P<0.01, *** P<0.001.

Abbreviations: SBP, systolic blood pressure; DBP, diastolic blood pressure; FPG, fasting plasma glucose; TG, triglycerides; TC, total cholesterol; HDLC, high-density lipoprotein cholesterol; LDLC, low-density lipoprotein cholesterol; CMRF, cardiometabolic risk factors; CI, Confidence interval.

a. Regression coefficients and 95% CIs from multivariate linear regression models. Adjusted for age, gender, educational degree, solitary status, and socio-economic degree of communities to which selected participants belonged.

b. Grouped by quartile intervals, the first LOBS interval representing the preponderance of pro-oxidants was used as a reference. Quartile 1, LOBS -6~-1; Quartile 2, LOBS 0; Quartile 3, LOBS 1; Quartile 4, LOBS 2.

**Table S6-2. Associations** **of the lifestyle-based oxidative balance score (quartile intervals) with the number of cardiometabolic risk factors and cardiometabolic biomarkers using logistic regression.**

|  | **SBP ^c^** | **DBP ^c^** | **FPG ^d^** | **TG ^e^** | **TC ^e^** | **HDLC ^e^** | **LDLC ^e^** | **Number of CMRFs** |
| --- | --- | --- | --- | --- | --- | --- | --- | --- |
| Quartile 1^b^ | / | / | / | / | / | / | / | / |
| Quartile 2 | 0.541** | 0.602* | 0.537 | 0.768 | 0.976 | 0.618 | 0.879 | 0.631* |
| 95%CI | 0.359, 0.813 | 0.356, 1.019 | 0.309, 0.932 | 0.462, 1.277 | 0.554, 1.722 | 0.367, 1.042 | 0.452, 1.709 | 0.440, 0.903 |
| Quartile 3 | 0.583* | 0.634 | 0.659 | 0.695 | 0.693 | 0.609 | 0.705 | 0.509*** |
| 95%CI | 0.372, 0.912 | 0.336, 1.195 | 0.349, 1.244 | 0.349, 1.244 | 0.350, 1.373 | 0.340, 1.091 | 0.322, 1.543 | 0.341, 0.759 |
| Quartile 4 | 0.624 | 0.582 | 0.475 | 0.550 | 0.573 | 0.553 | 0.555 | 0.460*** |
| 95%CI | 0.386, 1.008 | 0.275, 1.232 | 0.217, 1.040 | 0.285, 1.058 | 0.271, 1.211 | 0.286, 1.070 | 0.227, 1.356 | 0.301, 0.703 |
| P _trend_ | 0.023 | 0.910 | 0.270 | 0.053 | 0.096 | 0.032 | 0.156 | <0.001 |

* P<0.05, ** P<0.01, *** P<0.001.

Abbreviations: SBP: systolic blood pressure, DBP: diastolic blood pressure, FPG: fasting plasma glucose, TG: triglycerides, TC: total cholesterol, HDLC: high-density lipoprotein cholesterol, LDLC: low-density lipoprotein cholesterol, CMRF: cardiometabolic risk factors; CI, Confidence interval.

a. Odds ratios and 95% CIs from multivariate logistic regression models. Adjusted for age, gender, educational degree, solitary status, and socio-economic degree of communities to which selected participants belonged.

b. Grouped by quartile intervals, the first LOBS interval representing the preponderance of pro-oxidants was used as a reference. Quartile 1, LOBS -6~-1; Quartile 2, LOBS 0; Quartile 3, LOBS 1; Quartile 4, LOBS 2.

c. Blood pressure cutoffs: normal SBP, <140mmHg, abnormal SBP, ≥140 mmHg; normal DBP, <90 mmHg, abnormal DBP, ≥90 mmHg.

d. Fasting plasma glucose cutoffs: normal FPG, <7.0 mmol/L, abnormal FPG, ≥7.0 mmol/L.

e. Lipids/lipoproteins cutoffs: normal TG, <2.3mmol/L, abnormal TG, ≥2.3mmol/L; normal TC, <6.2mmol/L, abnormal TC, ≥6.2mmol/L; normal HDLC, >1.02mmol/L, abnormal HDLC, 1.0≤mmol/L; normal LDLC, <4.1mmol/L, abnormal LDLC, ≥4.1mmol/L.
